# Letrozole Potentiates Mitochondrial and Dendritic Spine Impairments Induced by ****β**** Amyloid

**DOI:** 10.1155/2013/538979

**Published:** 2013-07-17

**Authors:** P. K.-Y. Chang, S. Boridy, R. A. McKinney, D. Maysinger

**Affiliations:** Department of Pharmacology and Therapeutics, Faculty of Medicine, McGill University, 3655 Promenade Sir William Osler, Montreal, QC, Canada H3G 1Y6

## Abstract

Reduced estrogens, either through aging or postsurgery breast cancer treatment with the oral nonsteroidal aromatase inhibitor letrozole, are linked with declined cognitive abilities. However, a direct link between letrozole and neuronal deficits induced by pathogenic insults associated with aging such as beta amyloid (*Aβ*_1–42_) has not been established. The objective of this study was to determine if letrozole aggravates synaptic deficits concurrent with *Aβ*_1–42_ insult. We examined the effects of letrozole and oligomeric *Aβ*_1–42_ treatment in dissociated and organotypic hippocampal slice cultures. Changes in glial cell morphology, neuronal mitochondria, and synaptic structures upon letrozole treatment were monitored by confocal microscopy, as they were shown to be affected by *Aβ*_1–42_ oligomers. Oligomeric *Aβ*_1–42_ or letrozole alone caused decreases in mitochondrial volume, dendritic spine density, synaptophysin (synaptic marker), and the postsynaptic protein, synaptopodin. Here, we demonstrated that mitochondrial and synaptic structural deficits were exacerbated when letrozole therapy was combined with *Aβ*_1–42_ treatment. Our novel findings suggest that letrozole may increase neuronal susceptibility to pathological insults, such as oligomeric *Aβ*_1–42_ in Alzheimer's disease (AD). These changes in dendritic spine number, synaptic protein expression, and mitochondrial morphology may, in part, explain the increased prevalence of cognitive decline associated with aromatase inhibitor use.

## 1. Introduction 

Currently, aromatase inhibitors, leading to reduction of estradiol synthesis from testosterone, have been favoured in the treatment of breast cancer of postmenopausal women [1]. Letrozole is one such oral nonsteroidal aromatase inhibitor which prevents the aromatase from producing estrogens by competitive, reversible binding to the heme of its cytochrome P450 unit [2, 3]. Patients receiving letrozole have shown deficits in learning and memory [4]. However, it is still unclear if these functional impairments occur because of neurosteroid deficits and if they become exacerbated in the presence of an additional insult associated with aging, such as an excess of soluble, conformationally altered *Aβ*_1–42_. Such forms of *Aβ*_1–42_ can interact with synapses and cause pronounced degenerative changes, an observation made in brain tissue from postmortem Alzheimer's disease (AD) patients and animal models [5]. It has been well documented in AD that there are changes in neuronal and glial morphologies such as dendritic spine atrophy and increased glial growth [5, 6]. In contrast to these negative effects caused by *Aβ*_1–42_, it is well established that the neurosteroid estradiol can increase the number of dendritic spines and proteins in humans, as well as promoting synaptogenesis and cellular models of learning such as long-term potentiation (LTP) in hippocampal cultures [7, 8]. Despite these well-established positive effects of neuroestrogens in the CNS, 17-*β*-estradiol hormone therapy in postmenopausal women did not exhibit clear cognitive benefits [9–13]. These clinical studies did not provide conclusive evidence whether exogenous estrogen exposure in early menopause affects AD or cognitive aging [14] and to what extent (if any) peripheral estrogen in blood could play a role in patients or animal models of AD [15–17]. 

To investigate whether aromatase suppression in the CNS affects synaptic responses to pathological insults, we used confocal microscopy to examine the effect of letrozole and *Aβ*_1–42_ on two model systems, dissociated hippocampal neurons, and organotypic slice cultures. The slice cultures have a preserved neural circuitry, exhibit similar synaptic transmission as *in vivo*, and permit long-term studies allowing for the assessment of synapse morphology and function following pharmacological aromatase inhibition. The objective of the present study was to investigate the response of hippocampal neurons and glia to soluble *Aβ*_1–42_ species upon inhibition of CNS aromatase by letrozole. We have found significant decrease in neuronal mitochondrial volume, dendritic spine density, synapse number, and the synaptic protein, synaptopodin, in cultures that were either treated with letrozole or *Aβ*_1–42_ alone. With letrozole and *Aβ*_1–42_ co-treatment, the synaptic and mitochondrial deficits were further enhanced in the hippocampal neurons. These findings could explain why women on letrozole have a higher incidence of cognitive decline and may be more vulnerable to the detrimental effect of *Aβ*_1–42_.

## 2. Materials and Methods 

### 2.1. Preparation of *β*-Amyloid (*Aβ*_1–42_)

*Aβ*_1–42_ (Sigma-Aldrich, St. Louis, MO, USA) was dissolved in 5% NH4OH in Tris Buffered Saline and vortexed until fully dissolved (several minutes) at a concentration of 1 mM. Aliquots were stored at −80°C. Upon reconstitution, aliquots are dissolved directly in media prior to treatment at a final concentration of 1 *μ*M [18].

### 2.2. Preparation of Dissociated Neural Cell Cultures

All procedures for animal handling were carried out according to the guidelines of the Canadian Council on Animal Care (CCAC) and approved by the Animal Resource Committee of the School of Medicine, McGill University. Hippocampal tissues from mouse pups of 3–5 days old were dissected, trypsinized, dissociated, and incubated as described previously [19]. Briefly, hippocampi of P3–5mice were isolated, mechanically and enzymatically (0.25% trypsin; Gibco, Life Technologies Inc, Burlington, ON, Canada) dissociated, and plated onto coated (poly-l-ornithine, 100 *µ*g/mL (Sigma-Aldrich) and laminin, 0.587 *µ*g/mL (Invitrogen, Life Technologies Inc, Burlington, ON, Canada) glass coverslips and maintained at 37°C and 5% CO_2_ in a 24-well plate (Corning; Lowell, MA, USA). Culture medium consisted of neurobasal A (Invitrogen) supplemented with 2% (v/v) B-27 supplement (Invitrogen), 1% (v/v) PSN (Invitrogen), and 1 mM L-glutamine (Sigma-Aldrich). 

### 2.3. Pharmacological Treatments of Dissociated Neural Cell Cultures

Dissociated cells were treated on day *in vitro* (DIV) 8 with 1 *μ*M letrozole (Novartis, Dorval, QC, Canada) and/or 1 *μ*M *Aβ*_1–42_ (American Peptide Company, Sunnyvale, CA, USA) [20] according to the respective design outlined in the results section and corresponding figure legends.

### 2.4. Preparation of Slice Cultures

Hippocampal slices (400 *μ*m) from P6–8 transgenic mice expressing membrane-targeted MARCKS-eGFP under the Thy-1 promoter in a subpopulation of CA1 cells [21] were prepared as previously described using the roller-tube method [22]. Slice cultures were maintained in the incubator with a roller-drum for three weeks before experimentation to allow for spine maturation [23].

### 2.5. Pharmacological Treatments of Slice Cultures

Slice cultures were maintained for at least 3 weeks *in vitro *to allow for maturation prior to treatment. In addition, all treatments were done on sister cultures (slice cultures that were prepared from the same mice litter and handled the same way at the same time). To prevent the effects of estradiol known to be present in serum, which is normally included in the culture media, we placed our preparations in serum free media prior to testing the effects of *Aβ*_1–42_ or letrozole [24]. Following preincubation, cultures were then treated with media containing either, vehicle, *Aβ*_1–42_ (1 *μ*M), letrozole (1 *μ*M), or *Aβ*_1–42_ + letrozole for 24 or 72 hours. No media change was performed during the time course.

### 2.6. Immunocytochemistry for Dissociated Cells

Following treatments, the hippocampal cells were fixed in 4% formaldehyde for 10 min at room temperature (RT), and routine immunocytochemical protocol was followed using mouse monoclonal anti-GFAP (1 : 250; Molecular Probes, Life Technologies Inc, Burlington, ON, Canada), *β*-III tubulin (1 : 100; Molecular Probes), and Alexa488-conjugated goat anti-mouse IgG (1 : 800; Invitrogen). The samples were counterstained with Hoechst 33258 (Molecular Probes), mounted onto glass slides (Vectashield H-1000, Vector, Burlingame, CA, USA), and sealed with clear nail polish.

### 2.7. Mitochondrial Labeling and Morphological Status

Morphological status of mitochondria was determined by labelling live cells (treated or untreated) with 500 nM Mitotracker Deep Red FM (Molecular Probes) for 3 minutes and imaged with a confocal microscope at 63× (Zeiss LSM510, Carl Zeiss MicroImaging GmbH, Jena, Germany); 633 nm HeNe laser at 20% max excitation intensity. Z-stacks (10 slices, 0.33 *μ*m steps) were acquired of dendritic segments from 4–6 different neurons per coverslip, and 2-3 coverslips per condition were analyzed. 3D deconvolution of the raw stack was performed using Huygen's Deconvolution Software (Scientific Volume Imaging, Hilversum, The Netherlands) with maximum likelihood extrapolation, and images were further analyzed for volumetric measurements using Imaris (Bitplane, Zurich, Switzerland).

### 2.8. Image Acquisition and Quantification of Dendritic Spines

Following treatments, slice cultures were transferred to a temperature-controlled chamber (30°C) mounted on an upright confocal microscope equipped with W Plan-APOCHROMAT 63×/1.0 objective (Zeiss LSM710, Carl Zeiss MicroImaging GmbH) and continuously perfused with Tyrode solution containing (in mM): NaCl, 137; KCl, 2.7; CaCl_2_, 2.5; MgCl_2_, 2; NaHCO_3_, 11.6; NaH_2_PO_4_, 0.4; and glucose, 5.6 (pH 7.4). Secondary and tertiary dendritic branches from either apical or basal dendrites of CA1 pyramidal neurons were imaged using 488 nm argon-ion laser line. Spine classification was based on previously established criteria and separated into three main morphological subtypes, stubby, mushroom, and thin-type spines based on the ratio between the diameter and length of spine heads and necks [23].

### 2.9. Immunohistochemical Staining of Synaptic Proteins

Following treatment, hippocampal slice cultures were removed from the cover glass and fixed in 4% paraformaldehyde dissolved in 0.1 M phosphate buffer (PB) overnight at 4°C. Primary antibodies were incubated for five days at 4°C in the permeabilizing solution containing 0.4% Triton X 100. Rabbit monoclonal anti-synaptophysin primary antibody was used at 1 : 400 dilution (Zymed, CA, USA) and mouse monoclonal anti-synaptopodin primary antibody was used at 1 : 400 dilution (Abcam, MA, USA), as well. Secondary antibodies (Texas Red (Invitrogen); Cy-5 (Jackson ImmunoResearch Laboratories Inc., West Grove, PA, USA)) were prepared at 1 : 250 dilutions in 0.1 M PB containing 1.5% horse serum and incubated overnight at 4°C. Slices were then mounted with DAKO fluorescent mounting medium (Dako Canada, ON, Canada) onto microscope slides and imaged. Secondary and tertiary dendrites of CA1 pyramidal neurons were imaged using a Leica TCS SP2 scanhead (Leica Microsystems, Concord, ON Canada) on a Leica DM6000 B upright microscope (Leica Microsystems).

### 2.10. Statistical Analysis

Two-tailed, one-way ANOVA followed by Tukey's or Dunnet's multiple comparison tests or Student's *t*-tests is performed, and Bonferroni's correction is applied when appropriate. Results are expressed as mean ± SEM. All data analyses were performed blind.

## 3. Results

**Figure 1:**
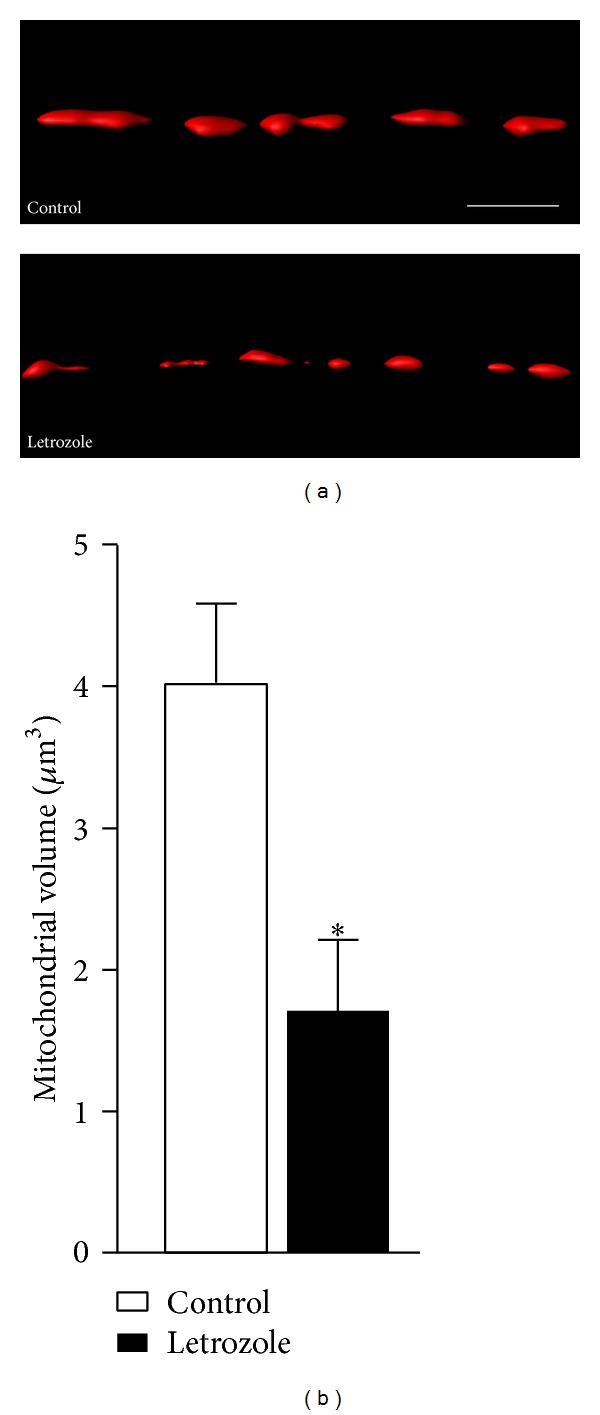
Mitochondrial impairments following *Aβ*_1–42_ and letrozole treatment. (a) Representative 3D reconstructions of MitoTracker Deep Red-positive mitochondria from hippocampal dendritic segments treated with letrozole (1 *μ*M) for 72 hours. Scale bar 5 *μ*m. (b) Quantifications of mitochondrial volumes. Significant differences in mitochondrial volume relative to control (4.03 ± 0.56 *μ*m^3^) were observed for letrozole (1.72 ± 0.49 *μ*m^3^, *P* = 0.015) and indicated by an asterix (*).

It has been previously shown that abnormalities in spine structure and composition are observed in response to aromatase inhibition; the present study considers the effects of soluble oligomeric *Aβ*_1–42_ on characterized dendritic spine subtypes and mitochondrial structures in hippocampal neurons exposed to letrozole. Estrogens mediate neuroprotective effects in part through maintenance of mitochondrial structure and metabolic function [25, 26], and, thus far, letrozole's effect on mitochondrial network plasticity has not been considered. Live dissociated mixed neural cultures treated for 24 hrs with 1 *μ*M letrozole were labelled with the mitochondrial-specific probe, MitoTracker Deep Red FM (see methods and materials). Sectioning of isolated hippocampal neuronal dendrites followed by 3D reconstruction of the resultant image stacks using Imaris software allowed for mitochondrial volume measurement acquisition and thus a readout of mitochondrial integrity, following aromatase inhibition (Figure 1(a)). We found that letrozole treatment resulted in mitochondrial fragmentation, measured as a significant reduction in volume (Figure 1(b), control, 4.03 ± 0.56 *μ*m^3^; letrozole, 1.72 ± 0.49 *μ*m^3^; *P* = 0.015), implying mitochondrial dysfunction. Next, we thought to address the question of how does blockade of estrogen synthesis affect neuronal circuitry.

**Figure 2:**
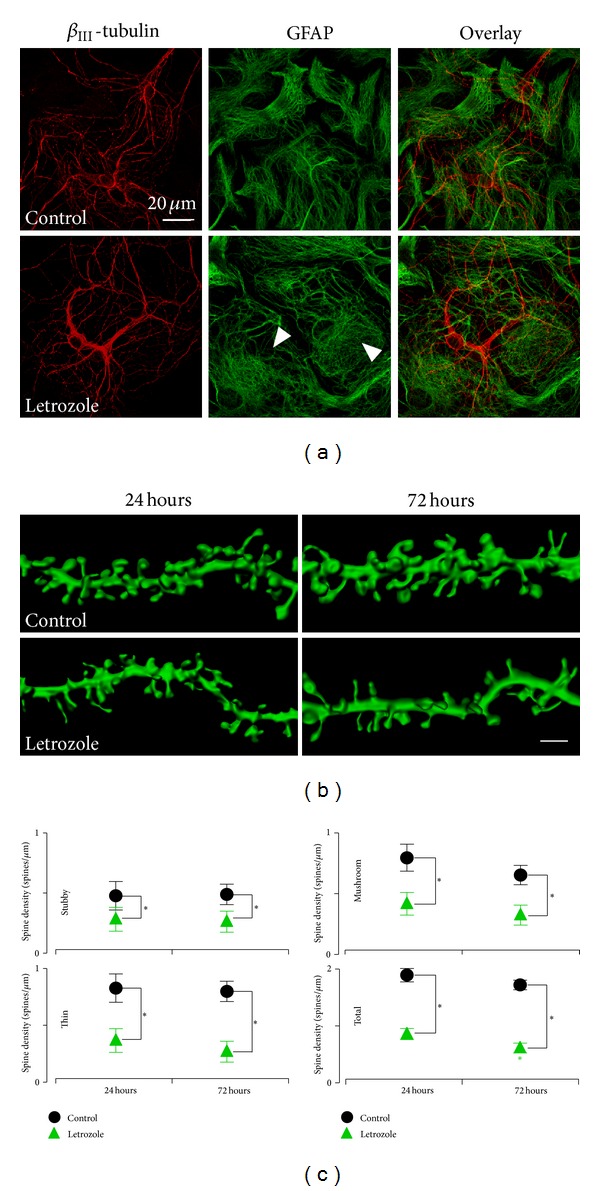
Hypertrophy of astroglia and a loss of dendritic spines following 3-day letrozole treatment. (a) Gender mixed hippocampal neural cells were isolated from 3-day old C57/BL6 mouse pups and fixed in 10% Formalin on DIV7. Shown is confocal images of immunocytochemically labelled hippocampal cell cultures with GFAP (astroglia, green) and *β*-III tubulin (neurons, red). Arrowheads outline astrocytic hypertrophy in letrozole-treated cultures after 72 hours. Scale bar, 20 *μ*m. (b) Representative 3D reconstructions of dendritic segments from organotypic hippocampal sister slice cultures that were treated with either control or letrozole (1 *μ*m)-containing media for either 24 or 72 hours. Scale bar, 2 *μ*m. (c) Quantification of the spine subtype densities following treatments. There is a significant decrease in the densities for all spine subtypes following letrozole treatment compared to control. 24-hour treatment, control, *n* = total dendritic segment lengths of 441 *μ*m from 10 cells in 4 cultures; letrozole, *n* = 433 *μ*m of dendrite from 9 cells in 4 cultures. 72-hour treatment, control, *n* = 500 *μ*m of dendrite from 12 cells in 4 cultures; letrozole, *n* = 489 *μ*m of dendrite from 11 cells in 4 cultures.

To this end, we examined the effects of estrogen synthesis inhibition on the morphological characteristics of neurons and glial cells using letrozole (Figure 2). Immunohistochemical analyses of colabelled neurons and glia from these mice using *β*-III tubulin as a neuronal marker and anti-glial fibrillary acidic protein (GFAP) as an astrocytic marker revealed profound changes in those cultures that were treated with letrozole (1 *μ*M) for 72 hours: primary cultures that were treated with control media have extensive neuronal arbors and normal astrocytic morphology while striking changes were revealed in the letrozole-treated cultures where hippocampal neurons were sparse, with thin dendritic extensions, coupled with massive astrocytic hypertrophy (Figure 2(a)). When the letrozole treatments were repeated using organotypic hippocampal slice cultures, a culture system with more intact neuronal circuitry, we observed a significant loss of dendritic spines following 24 and 72 hours of letrozole treatment. These findings suggest that the aromatase-mediated blockade of estradiol synthesis with letrozole adversely affects neuronal circuitry in the hippocampus.

As the densities of dendritic spines are known to be modulated by estrogens [8], we investigated the effect of letrozole on spine density and shape (Figure 2(b) and Supplementary Table 1). See Supplementary material available online at http://dx.doi.org/10.1155/2013/538979 Treatments for 24 hours with letrozole led to a significant reduction in the number of CA1 dendritic spines compared to control slices (Figure 2(c) and Supplementary Table 1, stubby, *P* = 2.1 × 10^−4^; mushroom, *P* = 1.0 × 10^−7^; thin, *P* = 2.1 × 10^−4^; total, *P* = 1.1 × 10^−6^). Given the observed decrease in spine density, we subsequently investigated whether the significant decrease in spine density was subtype dependent (thin, stubby, and mushroom), which could serve as a correlate of the number of glutamatergic *α*-amino-3-hydroxy-5-methyl-4-isoxazolepropionic (AMPA)-type receptors and synapse strength [27]. All subtypes of the spines were affected with the different treatments after 24 hours. Next, we determined if the dendritic spine loss following the treatment is time dependent, and we examined spine densities after 72 hours of letrozole treatment (Figure 2(c) and Supplementary Table 1, stubby, *P* = 7.6 × 10^−8^; mushroom, *P* = 3.3 × 10^−8^; thin, *P* = 1.8 × 10^−10^; total, *P* = 1.8 × 10^−10^). We found, at 72 hours, a continued loss of dendritic spines compared to treatments after 24 hours. These findings show that reduction of estradiol concentrations due to the aromatase inhibition affects all spine subclasses with the most marked reduction in mushroom and thin spines.

**Figure 3:**
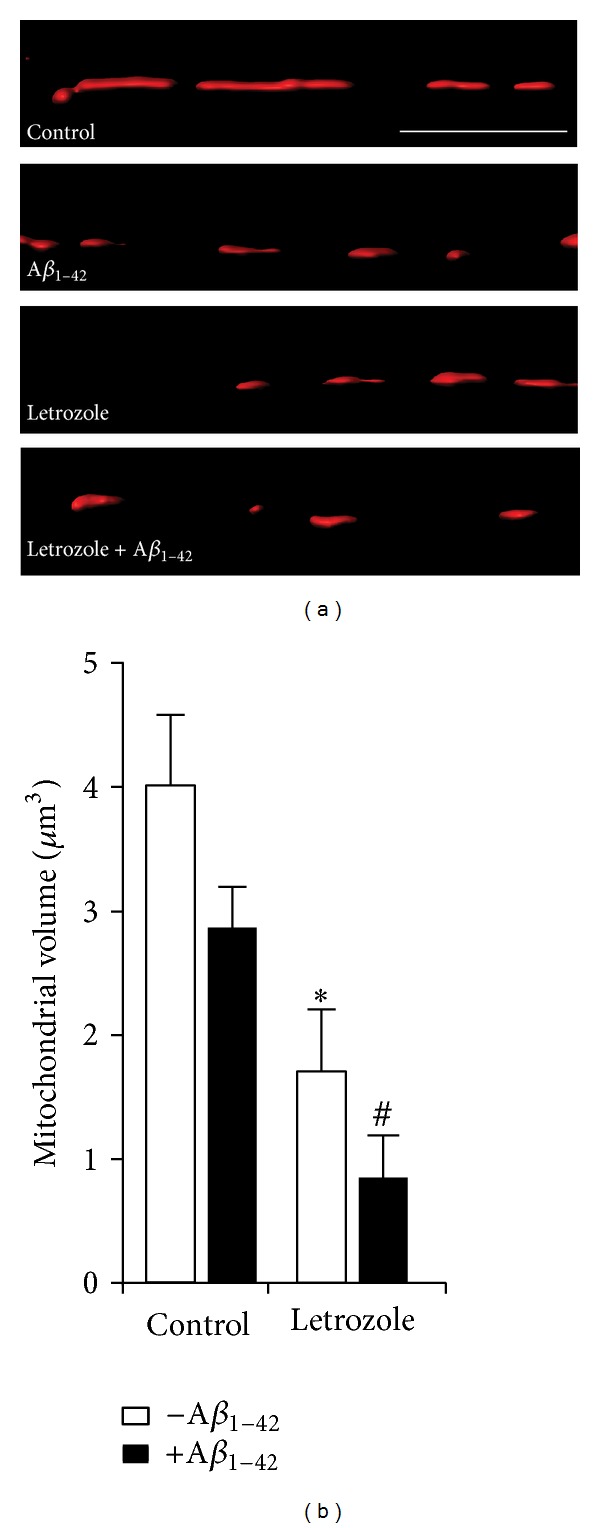
Letrozole and *Aβ*_1–42_ cause significant changes in mitochondrial morphology. (a) Representative 3D reconstructions of MitoTracker Deep Red FM-positive mitochondria from hippocampal dendritic segments treated with *Aβ*_1–42_ (1 *μ*M) for 24 hours in the presence and absence of 3-day letrozole (1 *μ*M) treatment. Scale bar, 5 *μ*m. (b) Quantifications of mitochondrial volumes. Significant differences in mitochondrial volume, compared to control cultures (4.03 ± 0.56 *μ*m^3^), were observed for letrozole- (1.72 ± 0.49 *μ*m^3^, *P* = 0.015) treated cultures, as indicated by (∗). Although there is no significant difference in mitochondrial volume between control and *Aβ*_1–42_ (*P* = 0.25), it is noteworthy that further reduction in mitochondrial volume relative to *Aβ*_1–42_ (2.88 ± 0.32 *µ*m^3^) was observed in letrozole + *Aβ*_1–42_ treated cultures (0.86 ± 0.33 *µ*m^3^, *P* = 3.37 × 10^−4^) and is signified by (#).

Given that letrozole alone caused mitochondrial impairments and synaptic deficits, we next determined whether it could exacerbate changes in response to soluble oligomeric *Aβ*_1–42_, an established mediator of neuronal deficits associated with AD pathology. First, we examined the effect of oligomeric *Aβ*_1–42_ in conjunction with letrozole treatment on neuronal mitochondrial integrity (Figure 3). Mixed dissociated neural cultures were treated with 1 *μ*M letrozole every 24 hours from DIV 6 to 8. On DIV 8, letrozole treatment was combined with oligomeric *Aβ*_1–42_ species (1 *μ*M) for a subsequent 24 hours, following which mitochondria were labelled and imaged as described above. As shown in Figure 3(b), we observed a trend towards a decrease (*P* = 0.26) in mitochondrial volume when the neuronal cultures were treated with *Aβ*_1–42_ alone. We found a further reduction of mitochondrial volume when the cultures were pretreated with letrozole prior to oligomeric *Aβ*_1–42_ species exposure compared to either letrozole (*P* = 0.17) or *Aβ*_1–42_ alone (*P* = 3.37 × 10^−4^). Our data suggest that letrozole treatment sensitizes neuronal mitochondria to alterations induced by *Aβ*_1–42_ oligomers. Next, we examined whether these treatments can also lead to changes in synaptic structures.

**Figure 4:**
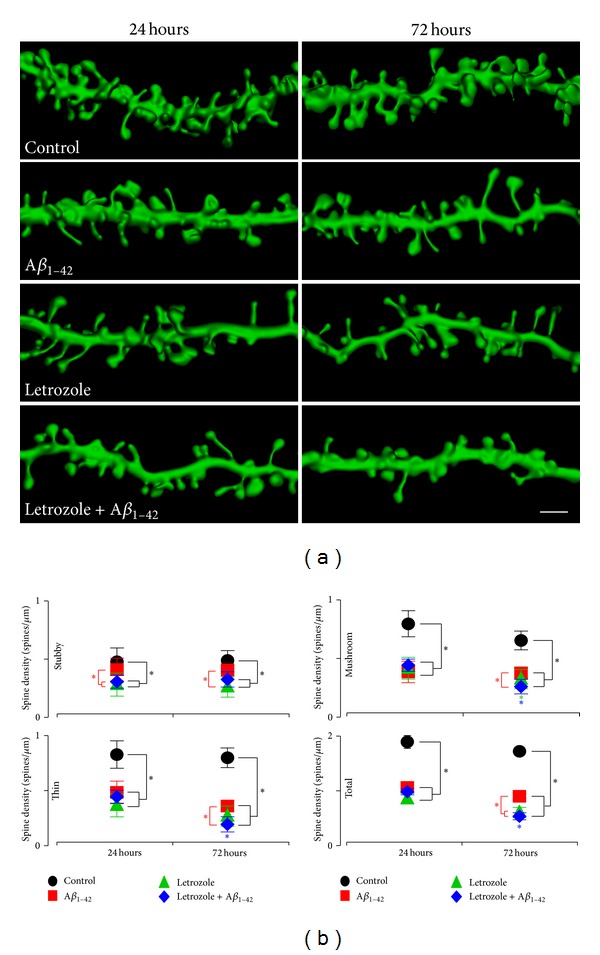
Letrozole and *Aβ*_1–42_ reduce the number of dendritic spines. (a) Representative 3D reconstructions of dendritic segments from sister cultures that were treated with either control, *Aβ*_1–42_ (1 *μ*M), and letrozole (1 *μ*M) or *Aβ*_1–42_ + letrozole (1 *μ*M) for 24 or 72 hours. Scale bar, 2 *μ*m. (b) Quantification of the spine subtype densities following treatments. There is a significant decrease in the total dendritic spine densities for *Aβ*_1–42_, letrozole, and *Aβ*_1–42_ + letrozole-treated cultures compared to control after 24 hours. When spine subtypes are examined, there are significant differences between control and treated cultures in mushroom and thin-type spines while spine density for stubby spines is only significantly different between letrozole and *Aβ*_1–42_ + letrozole compared to control and between *Aβ*_1–42_ and letrozole. Control, *n* = total dendritic segment lengths of 441 *μ*m from 10 cells in 4 cultures; *Aβ*_1–42_, *n* = 428 *μ*m of dendrite from 11 cells in 4 cultures; letrozole, *n* = 433 *μ*m of dendrite from 9 cells in 4 cultures; *Aβ*_1–42_ + letrozole, *n* = 431 *μ*m of dendrite from 10 cells in 4 cultures. There is a significant decrease in the total dendritic spine densities compared to control cultures after 72 hours as well. In total spine density, cultures treated with either letrozole or *Aβ*_1–42_ + letrozole had lower spine counts compared to cultures treated with *Aβ*_1–42_ alone. For mushroom and thin-type spines, cultures treated with both *Aβ*_1–42_ and letrozole had significantly lower spine densities compared to *Aβ*_1–42_ alone while stubby spine density was lower for letrozole-treated compared to *Aβ*_1–42_ alone. Control, *n* = 500 *μ*m of dendrite from 12 cells in 4 cultures; *Aβ*_1–42_, *n* = 465 *μ*m of dendrite from 10 cells in 4 cultures; letrozole, *n* = 489 *μ*m of dendrite from 11 cells in 4 cultures; *Aβ*_1–42_ + letrozole, *n* = 441 *μ*m of dendrite from 9 cells in 4 cultures. Only cultures treated with *Aβ*_1–42_ + letrozole had significant decrease in total spine densities between 24 and 72 hours of treatment. This difference is contributed by significant decrease in spine density in both mushroom and thin type spines.

Previously, it was shown that the addition of soluble oligomeric *Aβ*_1–42_ can cause a decrease in dendritic spine density [20]. Hence, we determined if the combined treatment with *Aβ*_1–42_ and letrozole exerts even greater impairments of mitochondrial integrity and synaptic structures. Treatments for 24 and 72 hours with either letrozole or *Aβ*_1–42_ alone lead to a significant reduction in the number of CA1 dendritic spines compared to control slices (Figure 4 and SI Table 2, *P* = 4.7 × 10^−5^, *P* = 1.1 × 10^−6^, resp.). The decrease in spines can be attributed to a drop in mushroom and thin-type spines (Figure 4 and Supplementary Table 2, mushroom: *Aβ*_1–42_, *P* = 1.7 × 10^−8^; letrozole, *P* = 1.0 × 10^−7^, thin: *Aβ*_1–42_, *P* = 2.1 × 10^−3^; letrozole, *P* = 2.1 × 10^−4^); while spine density for stubby spines is only significantly different between letrozole and *Aβ*_1–42_ + letrozole compared to control (letrozole, *P* = 2.1 × 10^−4^; *Aβ*_1–42_ + letrozole, *P* = 2.1 × 10^−4^) and between *Aβ*_1–42_ and letrozole (*P* = 0.014). In addition, treatments at 72 hours revealed a further reduction in dendritic spine densities in the slice cultures that were treated with both *Aβ*_1–42_ and letrozole compared to *Aβ*_1–42_ alone (Figure 4 and Supplementary Table 2,*P* = 1.9 × 10^−11^). In total spine density, cultures treated with either letrozole or *Aβ*_1–42_ + letrozole had lower spine count compared to cultures treated with *Aβ*_1–42_ alone (*P* = 0.036 and 3.3 × 10^−4^ resp.). For mushroom and thin-type spines, cultures treated with both *Aβ*_1–42_ and letrozole had significantly lower spine densities compared to *Aβ*_1–42_ alone (mushroom, *P* = 0.024; thin, *P* = 0.013) while stubby spine density was lower for letrozole-treated cultures compared to *Aβ*_1–42_ alone (*P* = 7.0 × 10^−3^). Furthermore, to examine whether the observed dendritic spine loss was indeed caused by estradiol depletion using letrozole, we treated the slice cultures with letrozole, *Aβ*_1–42_, and letrozole + *Aβ*_1–42_ in the presence of estradiol ( Supplementary Figure 1, Supplementary Tables 1 and 4, estradiol). We have found that cotreatment with estradiol successfully prevented the decrease in dendritic spine density caused by letrozole and/or *Aβ*_1–42_, as no change in spine densities was observed compared to control at 24 or 72 hours (24 hours: estradiol, *P* = 0.13; estradiol + letrozole, *P* = 0.38; estradiol + *Aβ*_1–42_, *P* = 0.22; estradiol + letrozole + *Aβ*_1–42_, *P* = 0.37; 72 hour, estradiol, *P* = 0.57; estradiol + letrozole, *P* = 0.18; estradiol + *Aβ*_1–42_, *P* = 0.69; estradiol + letrozole + *Aβ*_1–42_, *P* = 0.61). These data provide substantial evidence that letrozole can worsen neuronal deficits caused by oligomeric *Aβ*_1–42_.

**Figure 5:**
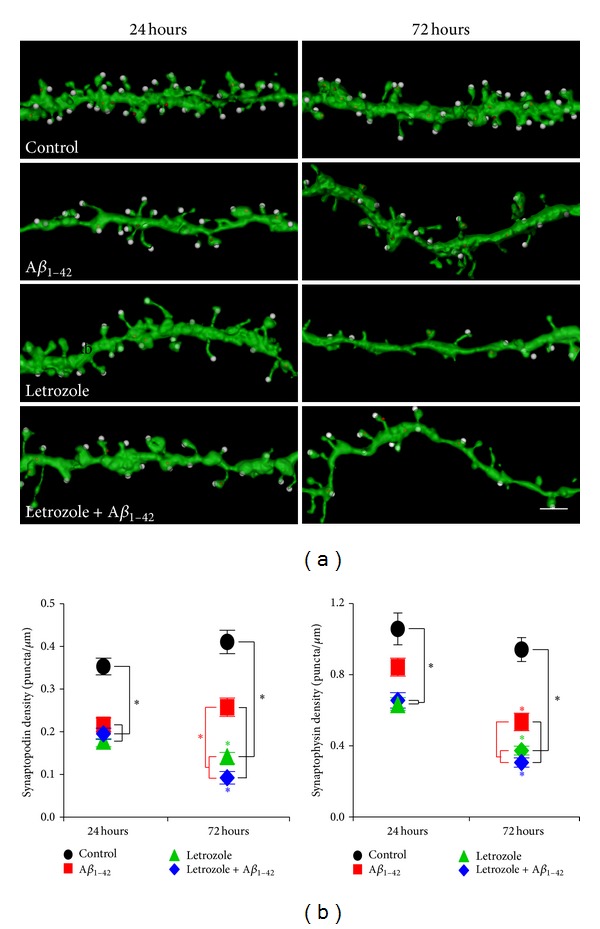
Letrozole and *Aβ*_1–42_ reduce synaptic proteins. (a) Representative 3D reconstructions of dendritic segments from sister cultures that were treated with either control, *Aβ*_1–42_ (1 *μ*M), and letrozole (1 *μ*m) or *Aβ*_1–42_ + letrozole (1 *μ*m) for 24 or 72 hours and immunostained for synaptophysin (white) and synaptopodin (red). Scale bar, 2 *μ*m. (b) Quantification of the densities of synaptopodin-positive puncta following treatments. There is a significant decrease in synaptopodin puncta after 24 and 72 hours of *Aβ*_1–42_, letrozole or *Aβ*_1–42_ + letrozole treatments compared to control conditions. After 72 hours, the number of synaptopodin puncta was significantly lower in letrozole and *Aβ*_1–42_ + letrozole-treated cultures compared to *Aβ*_1–42_ alone. When synaptopodin puncta densities were compared between 24- and 72-hour treated cultures there was a significant decrease only in letrozole and *Aβ*_1–42_ + letrozole-treated cultures after 72 hours. 24 hours: control, *n* = total dendritic segment lengths of 1041 *μ*m from 12 cells in 4 cultures; *Aβ*_1–42_, *n* = 633 *μ*m of dendrite from 8 cells in 4 cultures; letrozole, *n* = 952 *μ*m of dendrite from 10 cells in 4 cultures; *Aβ*_1–42_ + letrozole, *n* = 1007 *μ*m of dendrite from 10 cells in 4 cultures. 72 hours: control, *n* = total dendritic segment lengths of 559 *μ*m from 10 cells in 3 cultures; *Aβ*_1–42_, *n* = 472 *μ*m of dendrite from 9 cells in 4 cultures; letrozole, *n* = 838 *μ*m of dendrite from 9 cells in 4 cultures; *Aβ*_1–42_ + letrozole, *n* = 750 *μ*m of dendrite from 8 cells in 4 cultures. There is a significant decrease in synaptophysin puncta after 24 and 72 hours of *Aβ*_1–42_, letrozole, or *Aβ*_1–42_ + letrozole treatments compared to control conditions. After 72 hours, the number of synaptophysin puncta was significantly lower in letrozole and *Aβ*_1–42_ + letrozole-treated cultures compared to *Aβ*_1–42_ alone When synaptophysin puncta densities were compared between 24- and 72-hour treated cultures there was a significant decrease in *Aβ*_1–42_, letrozole, and *Aβ*_1–42_ + letrozole-treated cultures after 72 hours. 24 hours: control, *n* = total dendritic segment lengths of 1041 *μ*m from 12 cells in 4 cultures; *Aβ*_1–42_, *n* = 633 *μ*m of dendrite from 8 cells in 4 cultures; letrozole, *n* = 952 *μ*m of dendrite from 10 cells in 4 cultures; *Aβ*_1–42_ + letrozole, *n* = 1007 *μ*m of dendrite from 10 cells in 4 cultures. 72 hours: control, *n* = total dendritic segment lengths of 559 *μ*m from 10 cells in 3 cultures; *Aβ*_1–42_, *n* = 472 *μ*m of dendrite from 9 cells in 4 cultures; letrozole, *n* = 838 *μ*m of dendrite from 9 cells in 4 cultures; *Aβ*_1–42_ + letrozole, *n* = 750 *μ*m of dendrite from 8 cells in 4 cultures.

Although dendritic spines density was decreased after letrozole and *Aβ*_1–42_ treatment, it has been shown that the loss of spine does not necessary mean the loss of synapses [28]. In some instances, presynaptic terminals may be pulled back onto the dendrite to form asymmetric shaft synapses [28]. To answer the question if the observed loss of postsynaptic dendritic spines is accompanied by a loss of presynaptic terminals, we performed immunohistochemistry labelling of synaptophysin (presynaptic terminal marker). This presynaptic marker allows the determination of a loss or gain of presynaptic terminals when there is a loss (or gain) of dendritic spines induced by therapeutic treatments or insults to the CNS (Figure 5 and Supplementary Table 3). There was a significant decrease in synaptophysin puncta after 24 hours which became more pronounced at 72 hours of letrozole or *Aβ*_1–42_ + letrozole treatments compared to control conditions (24 hours: *Aβ*_1–42_, *P* = 6.1 × 10^−2^; letrozole,*P* = 7.3 × 10^−6^; *Aβ*_1–42_ + letrozole, *P* = 1.2 × 10^−5^; 72 hour, *Aβ*_1–42_, *P* = 4.0 × 10^−6^; letrozole, *P* = 4.0 × 10^−6^; *Aβ*_1–42_ + letrozole, *P* = 4.0 × 10^−6^). After 72 hours, the number of synaptophysin puncta was significantly lower in letrozole and *Aβ*_1–42_ + letrozole-treated cultures compared to *Aβ*_1–42_ or letrozole alone (letrozole, *P* = 0.043; *Aβ*_1–42_ + letrozole, *P* = 2.0 × 10^−3^). When synaptophysin puncta densities were compared between 24- and 72-hour treated cultures there was a significant decrease in *Aβ*_1–42_, letrozole, and *Aβ*_1–42_ + letrozole-treated cultures after 72 hours (*Aβ*_1–42_,*P* = 1.1 × 10^−4^; letrozole, *P* = 1.0 × 10^−7^; and *Aβ*_1–42_ + letrozole, *P* = 1.4 × 10^−8^). 

We next investigated if there was also a loss of synaptopodin, an actin-binding protein mainly found in a subgroup of mature spines. Synaptopodin is known to be downregulated upon exposure to estradiol [29]. It also plays a role in learning and memory; synaptopodin knockout animals have reduced LTP and have deficits in spatial learning [30]. We found that there is a change in synaptopodin staining after treatment with *Aβ*_1–42_, letrozole, and *Aβ*_1–42_ + letrozole (Figure 5 and SI Table 3). There was a significant decrease in synaptopodin puncta after 24 and 72 hours of treatments compared to control (Figure 5 and SI Table 3; 24 hours, *Aβ*_1–42_, *P* = 4.1 × 10^−6^; letrozole,*P* = 4.0 × 10^−6^; *Aβ*_1–42_ + letrozole, *P* = 4.0 × 10^−6^; 72 hours, *Aβ*_1–42_, *P* = 2.2 × 10^−4^; letrozole,*P* = 3.9 × 10^−6^; *Aβ*_1–42_ + letrozole, *P* = 3.9 × 10^−6^). After 72 hours, the number of synaptopodin puncta was significantly lower in letrozole and *Aβ*_1–42_ + letrozole-treated cultures compared to *Aβ*_1–42_ alone (letrozole, *P* = 1.2 × 10^−5^; *Aβ*_1–42_ + letrozole, *P* = 3.9 × 10^−6^). When synaptopodin puncta densities were compared between 24- and 72-hour treated cultures, there was a significant decrease in *Aβ*_1–42_ (*P* = 4.1 × 10^−6^), letrozole (*P* = 4.0 × 10^−6^), and *Aβ*_1–42_ + letrozole (*P* = 4.0 × 10^−6^)-treated cultures after 72 hours. Difference between controls was not significant (*P* = 0.09). Decreases in estrogen levels reduced the number of synaptic connections, and this was more pronounced in the presence of *Aβ*_1–42_. 

## 4. Discussion

Chronic estrogen deficit, similar to that experienced by menopausal women, is a recognized risk factor for AD [31]. The neurological deficits known to result from estrogen deficiency are accompanied by morphological deterioration of postsynaptic spines and functional impairments of mitochondria. We have now shown similar pathological features in an animal model with estrogen deficiency [32] and in neural cells of normal animals with estrogen deficiency induced by letrozole, a therapeutic agent for breast cancer. However, in both instances, the more marked dendritic spine degeneration was produced only when letrozole treatment was together with oligomeric *Aβ*_1–42_.

Dendritic atrophy and astrocytic hypertrophy are common observations in many neurological disorders [6], including AD [33]. Although it was very clear that both circulating and brain estrogens were reduced in animal models and can lead to learning deficits, the contribution of neuroestrogens versus circulating estradiol could not be determined [15, 34]. To circumvent this, we used hippocampal organotypic cultures treated with the aromatase inhibitor, letrozole. This experimental paradigm allowed us to control the concentration and duration of aromatase inhibition and estrogen deficit. Moreover, this model is optimal for studying neuronal connections and glial input in a spatiotemporal (longitudinal) manner, in parallel with studies of mitochondrial responses to local estrogen deficits. 

Functional and morphological impairment of synaptic mitochondria was associated with accumulation of *Aβ*_1–42_ in AD animal models [35, 36]. How does this relate to synaptic functions? Neuronal activity was shown to increase mitochondrial fission and entry of mitochondria into dendritic spines [37]; however fission is also initiated to sequester dysfunctional mitochondrial components and to facilitate apoptosis associated with neurodegenerative changes [38]. Recently, it has been suggested that mitochondria associated with dendritic spines may play an important role in regulating AMPA receptor trafficking, which is altered in the presence of *Aβ*_1–42_ oligomers [5, 39]. We were interested in whether changes in mitochondrial morphology under conditions of reduced estrogen synthesis in the presence of *Aβ*_1–42_ oligomers, were associated with synaptic deficits. We noted a significant decrease in mitochondrial volume, suggesting fission after *Aβ*_1–42_, letrozole, or combined treatments with both. These observations are therefore in line with our present understanding of how synaptic degeneration relates to mitochondrial impairment. 

Our results also suggest that estradiol may play a role in regulating the balance between the two opposing processes of mitochondrial fission and fusion. Dynamics between these two processes maintains mitochondrial homeostasis in healthy cells but is disrupted in AD [40, 41]. Findings reported in this study are in accordance with recent findings indicating a role for *Aβ*_1–42_ in the regulation of mitochondrial fission in AD [40, 42]. Further emphasizing the importance of mitochondria in spine maintenance, impairment of mitochondrial transport to synapses by *Aβ*_1–42_ has been reported to contribute to AMPAR removal and trafficking defects leading to synaptic inhibition [39].

In order to further understand how these disturbances in mitochondrial dynamics affect neuronal plasticity and function, we next investigated dendritic spine morphology, density, and synaptic proteins, following similar treatments. We examined changes in synaptophysin, a presynaptic marker, and synaptopodin, an actin binding protein, mainly found in a subgroup of mature spines and is regulated by estradiol [29]. The precise role of synaptopodin in hippocampal neurons is still unclear but has been shown to be important in learning and memory and in glutamate functions [30]. Our data show a significant reduction of pre- and postsynaptic components and synaptopodin with *Aβ*_1–42_ and letrozole treatment.

**Figure 6:**
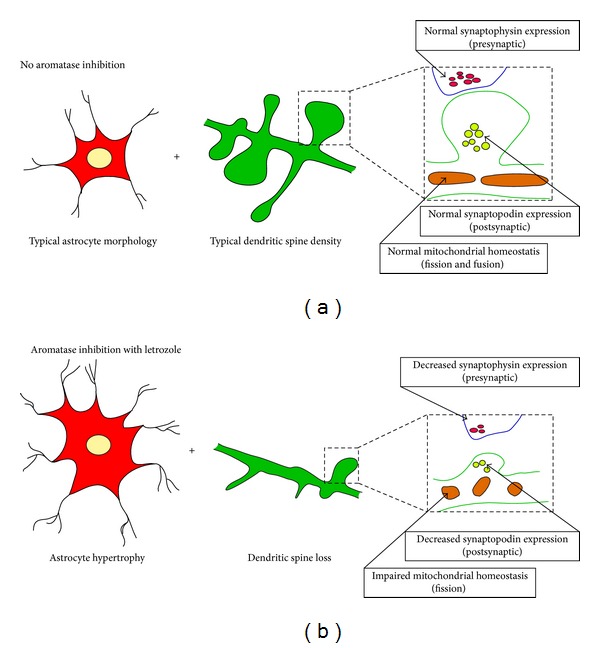
Summarising schematic: aromatase inhibition leads to synaptic and mitochondrial deficits. (a) Aromatase-mediated conversion of testosterone to estrogen in astrocytes serves as a local reservoir of neuroestrogens to promote maintenance of dendritic spines and synaptic mitochondrial integrity. (b) Aromatase inhibition by letrozole leads to spine loss, pre- and postsynaptic protein deficits, and compromised mitochondrial integrity.

Taken together, present results suggest that the neurological deficits seen in women on letrozole therapy for breast cancer could result, at least in part, from the therapy-induced hippocampal estrogen deficiency (Figure 6). Herein, we demonstrate that dendritic spine and mitochondrial deteriorations are particularly prominent in states of central hypoestrogenicity (Figure 6, lower half) and can further increase hippocampal susceptibility to spine/synaptic loss or mitochondrial functional impairment induced by a pathological insult. An important outcome of the current studies is the recognition that caution must be exercised in the use of letrozole as a therapy for breast cancer, and that account must be taken especially of the age of the female patient and/or presence of other recognised risk factors for AD. Given that letrozole is highly lipophilic and can cross the blood brain barrier, its incorporation into a nanodelivery system to prevent its access to the brain and at the same time enhance accumulation in the tumor warrants consideration.

## Supplementary Material

Slice cultures were maintained for at least 3 weeks *in vitro* to allow for maturation prior to treatment. In addition, all treatments were done on sister cultures (slice cultures that were prepared from the same mice litter and handled the same way at the same time). To prevent effects of estradiol present in serum, we pre-incubated our preparations in serum free media prior to any treatment. Following overnight pre-incubation in serum free media, the cultures were then treated with estradiol (100 nM)), estradiol (100 nM) + A*β*_1-42_ (1 *µ*M), letrozole (1 *µ*M) + estradiol (100 nM), or A*β*_1-42_ (1 *µ*M) + letrozole (1 *µ*M) + estradiol (100 nM) for 24 and 72 hours.
