# Supplementary material for: Letrozole Potentiates Mitochondrial and Dendritic Spine Impairments Induced by β Amyloid
Source: J Aging Res. 2013 Jul 17;2013:538979. doi: 10.1155/2013/538979 (PMC3730361; doi:10.1155/2013/538979)
Supplement: Supplementary file 1 — Slice cultures were maintained for at least 3 weeks in vitro to allow for maturation prior to treatment. In addition, all treatments were done on sister cultures (slice cultures that were prepared from the same mice litter and handled the same way at the same time). To prevent effects of estradiol present in serum, we pre-incubated our preparations in serum free media prior to any treatment. Following overnight pre-incubation in serum free media, the cultures were then treated with estradiol (100 nM)), estradiol (100 nM) + Aβ 1-42 (1 µM), letrozole (1 µM) + estradiol (100 nM), or Aβ 1-42 (1 µM) + letrozole (1 µM) + estradiol (100 nM) for 24 and 72 hours. [file 538979.f1.pdf]

Supplementary information:

Table 1: Dendritic spine density after 24 or 72 hours of control and letrozole-treated cultures

	Spine density (spine/µm)			
Spine subtype	24 hours		72 hours	
	control	letrozole	control	letrozole
Stubby	0.43 ± 0.029	0.26 ± 0.018	0.42 ± 0.021	0.23 ± 0.019
Mushroom	0.72 ± 0.033	0.38 ± 0.032	0.59 ± 0.033	0.29 ± 0.026
Thin	0.73 ± 0.055	0.35 ± 0.055	0.72 ± 0.049	0.26 ± 0.054
Total	1.88 ± 0.10	0.99 ± 0.084	1.73 ± 0.073	0.78 ± 0.075

The data used to generate the contents of this table was also used for statistical analysis. Statistical analysis was done using two sample Student's *t*-test as described in the "Materials and Method" section. The calculated p-values are displayed and incorporated into the main text in the "Results" section. Data presented here correspond to Figure 2B and C.

Table 2: Dendritic spine density after 24 or 72 hours of control, letrozole, Aβ, or letrozole+Aβ-treated cultures.

	Spine density (spine/µm)							
Spine subtype	24 hours				72 hours			
	control	letrozole	$A\beta_{1-42}$	letrozole+ $A\beta_{1-42}$	control	letrozole	$A\beta_{1-42}$	letrozole+ $A\beta_{1-42}$
Stubby	0.43±0.029	0.26±0.018	0.37±0.025	0.28±0.022	0.42±0.021	0.23±0.019	0.36±0.029	0.29±0.028
Mushroom	0.72±0.033	0.38±0.032	0.35±0.031	0.40±0.022	0.59±0.033	0.29±0.026	0.34±0.020	0.23±0.029
Thin	0.73±0.055	0.35±0.055	0.45±0.063	0.42±0.032	0.72±0.049	0.26±0.054	0.33±0.027	0.20±0.030
Total	1.88±0.10	0.99±0.084	1.17±0.084	1.10±0.047	1.73±0.073	0.78±0.075	1.02±0.038	0.72±0.056

The data used to generate the contents of this table was also used for statistical analysis Statistical analysis was done using Two-tailed, One-way ANOVA followed by Tukey's multiple comparison test as described in the "Materials and Method" section. The calculated p-values are displayed and incorporated into the main text in the "Results" section. Data presented here correspond to Figure 4.

Table 3: Immunostaining punta density after 24 or 72 hours of control, letrozole, Aβ, or letrozole+-treated cultures

	Spine density (spine/µm)							
Synaptic protein	24 hours				72 hours			
	control	letrozole	$A\beta_{1-42}$	letrozole+ $A\beta_{1-42}$	control	letrozole	$A\beta_{1-42}$	letrozole+ $A\beta_{1-42}$
Synaptopodin	0.35±0.020	0.17±0.0096	0.22±0.016	0.19±0.013	0.41±0.028	0.14±0.013	0.26±0.022	0.092±0.015
Synaptophysin	1.06±0.090	0.64±0.035	0.84±0.050	0.65±0.043	0.94±0.067	0.37±0.025	0.53±0.049	0.31±0.026

The data used to generate the contents of this table was also used for statistical analysis Statistical analysis was done using Two-tailed, One-way ANOVA followed by Tukey's multiple comparison test as described in the "Materials and Method" section. The calculated p-values are displayed and incorporated into the main text in the "Results" section. Data presented here correspond to Figure 5.

Table 4: Dendritic spine density after 24 or 72 hours of estradiol, letrozole, Aβ, or letrozole+Aβ-treated cultures.

Spine subtype	Spine density (spine/μm)							
	24 hours				72 hours			
	estradiol	estradiol+ letrozole	estradiol+ $A\beta_{1-42}$	estradiol+ letrozole+ $A\beta_{1-42}$	estradiol	estradiol+ letrozole	estradiol+ $A\beta_{1-42}$	estradiol+ letrozole+ $A\beta_{1-42}$
Stubby	0.36±0.022	0.37±0.026	0.39±0.036	0.40±0.030	0.40±0.027	0.40±0.020	0.43±0.024	0.42±0.021
Mushroom	0.64±0.030	0.66±0.027	0.63±0.038	0.74±0.027	0.62±0.039	0.55±0.035	0.53±0.032	0.65±0.029
Thin	0.66±0.067	0.75±0.040	0.69±0.054	0.83±0.035	0.67±0.033	0.65±0.044	0.75±0.031	0.73±0.042
Total	1.66±0.094	1.78±0.059	1.71±0.089	1.78±0.065	1.68±0.050	1.60±0.075	1.70±0.051	1.78±0.061

The data used to generate the contents of this table was also used for statistical analysis Statistical analysis was done using Two-tailed, One-way ANOVA followed by Tukey's multiple comparison test as described in the "Materials and Method" section. The calculated p-values are displayed and incorporated into the main text in the "Results" section. Data presented here correspond to SI Figure 1.

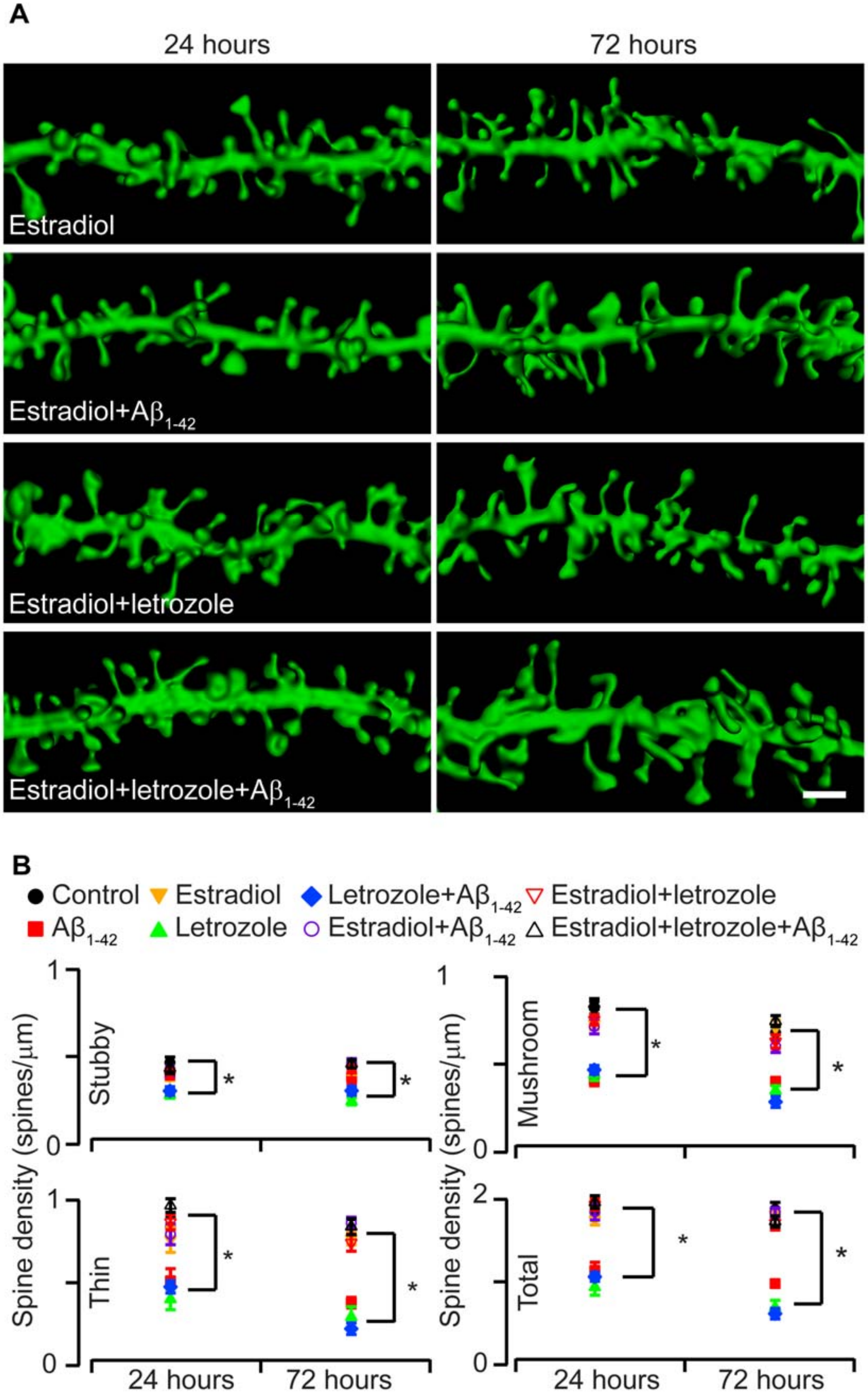
A
24 hours
72 hours
Estradiol
Estradiol+Aβ1-42
Estradiol+letrozole
Estradiol+letrozole+Aβ1-42
B
Control
Estradiol
Letrozole+Aβ1-42
Estradiol+letrozole
Aβ1-42
Letrozole
Estradiol+Aβ1-42
Estradiol+letrozole+Aβ1-42
Stubby
Mushroom
Thin
Total
Spine density (spines/μm)
24 hours
72 hours
*

Figure 1: Estradiol co-treatment prevents dendritic spine loss caused by letrozole and/or $A\beta_{1\text{-}42}$.

A, 3D-reconstructions of dendrites from sister-cultures that were treated with either estradiol (100 nM), estradiol (100 nM) + Aβ (1 µM), letrozole (1 µM) + estradiol (100 nM), or Aβ (1 µM) +letrozole (1 µM) +estradiol (100 nM) for 72 hours. Scale bar, 2 µm. **B**, Quantification of the spine subtype densities following treatments. There is a significant decrease in the total dendritic spine densities for $A\beta_{1\text{-}42}$, letrozole, and Letrozole+$A\beta_{1\text{-}42}$-treated cultures compared to control and cultures treated in the presence of estradiol after 24 hours. Estradiol prevented the dendritic spine loss caused by $A\beta_{1\text{-}42}$, letrozole, and letrozole+$A\beta_{1\text{-}42}$ at both 24 and 72 hours. 24 hours Control, n = total dendritic segment lengths of 441 µm from 10 cell in 4 cultures; estradiol, n = 625 µm of dendrite from 12 cell in 5 cultures; estradiol+$A\beta_{1\text{-}42}$, n = 725 µm of dendrite from 13 cell in 4 cultures; estradiol+letrozole, n = 862 µm of dendrite from 10 cell in 6 cultures; estradiol+letrozole+$A\beta_{1\text{-}42}$, n = 645 µm of dendrite from 11 cell in 5 cultures. 72 hours: Control, n = 500 µm of dendrite from 12 cell in 4 cultures; estradiol, n = 710 µm of dendrite from 10 cell in 5 cultures; estradiol+$A\beta_{1\text{-}42}$, n = 1016 µm of dendrite from 14 cell in 5 cultures; estradiol+letrozole, n = 688 µm of dendrite from 12 cell in 6 cultures; estradiol+letrozole+$A\beta_{1\text{-}42}$, n = 859 µm of dendrite from 10 cell in 4 cultures.
